# Food bioactive componts, a possible adjuvant for H.pylori eradication

**DOI:** 10.22088/cjim.8.2.131

**Published:** 2017

**Authors:** Soheil Ebrahimpour, Haleh Esmaeili, Reza Ghadimi

**Affiliations:** 1 Infectious Diseases and Tropical Medicine Research Center, Babol University of Medical Sciences, Babol, Iran.; 2 Social Determinants of Health Research Center, Health Research Institute, Babol University of Medical Sciences, Babol, Iran.

**Keywords:** Helicobacter pylori, adjuvant therapy, dietary bioactive components

**Dear Editor,**

*Helicobacter pylori (H. pylori)* is a Gram-negative bacillus. Since its discovery in the early eighties by warren and marshall, for the first time in the history of medicine H. pylori was isolated from human gastric biopsy specimens (1). Many studies have presented that *H. pylori* can be isolated from the oral cavity and salivary secretions. Colonization of the gastric pits in the stomach by this brachium is a serious risk factor for peptic ulcers and stomach cancer (2). 

Several antibiotic regimens have been assessed for *H. pylori *therapy. Despite that, few regimens have shown high eradication rates. Therapeutic regimens of *H. pylori* infection are usually based on at least two types of antibiotics (imidazoles, macrolides and amoxicillin) combined with a double dose of the proton-pump inhibitor (omeprazole or pantoprazole). The problem of eradication therapy is the potentially undesirable increasing resistance of *H. pylori* to the commonly used antibiotics. Moreover, eradication therapy is associated with some side effects. Long-term therapy with the antibiotic can result in pervasive alterations in gut flora and lead to susceptibility infections. So, the development of the adjuvant therapy for the eradication of *H. pylori* which also prevent adverse side effects would be a valuable item.

 Accordingly, different potential adjuvant therapies for *H. pylori* have been considered. The major research on alternative therapies contains foods like plant origin, probiotics, and polysaccharides. Some compounds from medicinal plants with anti-*H.pylori *activity consist of polyphenolic, sulforaphane, flavonoids, carvacrol, tannins, quercetin and β-hydrastine (3, 4). Green tea contains polyphenols, which hamper the growth of bacteria. Some studies have shown that drinking green tea can decrease *H. pylori *colonization during standard treatments (5). Broccoli sprouts (high in sulphorafane), cruciferous vegetables include kale, cabbage, cauliflower, brussels sprouts, red-headed cabbage, and radishes (high in isothiocyanates) can diminish the *H. pylori* growth (6). Apples contain flavonoids that defend the lining materials of the stomach against *H.pylori* infection (7). Honey exhibits potent in vitro bacteriostatic activity against *H. pylori* and inhibits urease activity. Antibacterial activity of honey is attributable to its hydrogen peroxide content (5). Probiotic bacteria can modulate *H. pylori* activity by either immunological (reduction of IL-1 and IL-6) or non-immunological mechanisms (competition with potential microorganisms) (3). Therefore, the use of antibiotics as first-line therapies may be appropriate for the eradication of *H.pylori*, but the results confirm the medicinal properties of some foods and compounds as a new adjuvant therapy for *H.pylori* eradication. Moreover, further studies should be required to support their use.
